# Expanding the Molecular-genetic Spectrum of Canalicular Adenoma-like Subtype of Pleomorphic Adenoma of Salivary Glands

**DOI:** 10.1097/PAS.0000000000002377

**Published:** 2025-03-04

**Authors:** Natálie Klubíčková, Frederica Loghides, Mari F.C.M. van den Hout, Valérie Costes-Martineau, Gerardo Ferrara, Miguel Rito, Veronika Hájková, Petr Grossmann, Petr Šteiner, Inka Kovářová, Michal Michal, Ilmo Leivo, Alena Skálová

**Affiliations:** * Department of Pathology, University Hospital and Faculty of Medicine in Pilsen, Charles University, Czech Republic; ‡ Biomedical Center, Faculty of Medicine in Pilsen, Charles University, Czech Republic; † Bioptical Laboratory, Ltd., Pilsen, Czech Republic; § Department of Anatomical Pathology, Canterbury District Health Board, Christchurch Hospital, Christchurch, New Zealand; ∥ Department of Pathology, School for Oncology and Reproduction (GROW), Maastricht University Medical Center, Maastricht, The Netherlands; ¶ Department of Pathology, University Hospital of Montpellier, Montpellier, France; # REFCORpath, France; ** Anatomic Pathology and Cytopathology Unit, Istituto Nazionale Tumori IRCCS Fondazione ‘G. Pascale’, Via Mariano Semmola, Naples, Italy; †† Serviço de Anatomia Patológica, Instituto Português de Oncologia de Lisboa, Portugal; ‡‡ Faculdade de Medicina, Instituto de Anatomia Patológica, Universidade de Lisboa, Lisbon, Portugal; §§ Institute of Biomedicine, Pathology, University of Turku and Department of Pathology, Turku University Hospital, Turku, Finland

**Keywords:** salivary glands, canalicular adenoma-like subtype of pleomorphic adenoma, *HMGA2::WIF1*, apocrine transformation, epithelial-myoepithelial carcinoma

## Abstract

Canalicular tumors of the salivary glands have recently emerged as an entity characterized by distinct morphology and recurrent *HMGA2* gene rearrangement. In this study, we analyzed 40 cases intending to elucidate their features further. The monophasic or biphasic tumors exhibited a growth pattern of interconnected anastomosing trabeculae and canaliculi, accompanied by a classical pleomorphic adenoma in one-third of the cases. Invasive growth into surrounding adipose tissue was revealed in one case which was, therefore, diagnosed as epithelial-myoepithelial carcinoma. Although the tumor cells uniformly expressed HMGA2 protein in all cases, cytokeratin 7, S100 protein, and SOX10 displayed either diffuse positivity or highlighted the luminal and abluminal cell populations, respectively. Areas with morphological oncocytoid change and AR-immunopositivity of luminal cells were seen in 13/14 (93%) of tested biphasic cases. *HMGA2* rearrangement was detected by RNA-sequencing in 30 cases. The most common alteration was an *HMGA1::WIF1* fusion, but several novel or rare fusion partners were identified, including *ARID2*, *FHIT*, *MSRB3* and its antisense variant *MSRB3-AS1*, *IFNG-AS1*, and the long intergenic region *LINC02389*. In addition, FISH revealed *HGMA2* break-apart in the remaining 10 cases where targeted sequencing failed to detect any alteration or where RNA sequencing could not be performed. Notably, the loss of the 3’-untranslated region of *HMGA2* emerges as the common denominator for the described rearrangements, possibly disrupting its negative regulation by small regulatory RNAs. Awareness of this lesion ensures appropriate diagnosis and clinical management, especially with regard to the possibility of malignant transformation described in this and previous studies.

Although rearrangement of the *HMGA2* gene has been repeatedly reported in pleomorphic adenoma (PA) and carcinoma ex PA, Agaimy et al^1^ recently described 28 cases of morphologically unique tumors, exhibiting a distinctive canalicular and trabecular growth pattern, common p40 and p63 immunonegativity, and *HMGA2* fusions detected in 14/16 analyzable cases. Thirteen of these cases also included a variably abundant component of a classical PA. Follow-up information was available in 9 cases with a median length of 16 months, with no adverse outcomes including recurrence.

Katabi et al^2^ described 8 tumors occurring mostly in major salivary glands and harboring an *HMGA2::WIF* fusion, as well as 2 tumors with alternate *HMGA2* rearrangements (*HMGA2::LOC105373146* and truncated *HMGA2* with exons 1 to 3 retained, respectively). The majority of these tumors grew in a distinctive pattern of interconnected canaliculi and trabeculae of monolayered or multilayered epithelium endowed in hyalinized or myxoid stroma, whereas 1 case was described as myoepithelial-rich PA. Three cases in this series were malignant, and 1 of these patients died of disease. All tested cases were immunopositive for S100 and CK7, whereas p40 was completely negative and p63 showed mostly weak and focal staining. In addition, in a literature review of *HMGA2::WIF*-rearranged tumors authored by Katabi et al,^2^ most cases exhibited canalicular architecture and a similar immunoprofile, and a classical PA component was present in about half of the cases. Up to a fifth (21%) of tumors were reportedly malignant. However, given the fact that molecular-genetic analysis is more likely to be done only in unusual, challenging cases of PA or in cases where other, possibly malignant entities are considered, the rate of malignant tumors arising in all cases of the canalicular variant of PA described here is probably much lower.

A recent study by Alsugair et al^3^ described 46 cases of PA with *HMGA2* rearrangements, most prevalently *HMGA2::WIF* fusions. The morphology of the described cases ranged from conventional PA with myxochondroid stroma and biphasic cell population to canalicular adenoma-like subtype of PA, with 9 cases exhibiting a mixed growth pattern of both components.

Building upon this background, the present study aims to provide further histomorphologic and molecular-genetic data on 40 cases of the emerging canalicular adenoma-like subtype of PA of the salivary glands. All cases presented herein were tested by molecular-genetic techniques, revealing novel and rare partners to *HMGA2* apart from the canonical *HMGA2::WIF* fusion. Recognition of this PA subtype ensures appropriate management. Although the neoplasm usually exerts indolent behavior, our study and previous reports suggest a very rare occurrence of malignant transformation. An association of *MDM2* amplification in 4 HMGA2-overexpressing carcinomas ex PA with particular gear-like nuclear atypia was recently reported,^4^ prompting further analysis of the phenomenon in our study.

## MATERIALS AND METHODS

### Case Selection

For our study, 62 cases of salivary gland tumors with canalicular adenoma-like morphology were retrieved from the consultation registry of one diagnostic histopathologic laboratory. The registry contains 7932 cases of rare salivary gland cases received for a consultation over the period of 22 years. In 30/62 cases, *HMGA2* rearrangements were detected by FISH or targeted RNA-sequencing, and these cases were further analyzed and described in this study. In addition, 10 cases with a corresponding morphology, immunoprofile and genetic background were added prospectively while the study was being conducted. The morphology and immunohistochemical profiles were reviewed by 3 pathologists (N.K., F.L., and A.S.). The majority of cases were received for consultation; treatment and outcome data were therefore available in 7 cases only.

### Histology and Immunohistochemistry

For conventional microscopy, the excised tissues were fixed in formalin, processed routinely, embedded in paraffin (FFPE), cut, and stained with hematoxylin and eosin. For the HMGA2, PLAG1, S100, SOX10, CK7, p63 and p40, AR, and MDM2 immunohistochemistry, the 4-μm-thick sections cut from paraffin blocks were processed in accordance with institutional standards on BenchMark ULTRA (Ventana Medical Systems). All primary antibodies used in this study are summarized in Table 1. Visualization was performed using the ultraView Universal DAB Detection Kit (Roche) and ultraView Universal Alkaline Phosphatase Red Detection Kit (Roche). The slides were counterstained with Mayer hematoxylin. Appropriate controls were used.

**TABLE 1:** Antibodies Used for the Immunohistochemical Examination.

Marker	Clone	Company	Dilution
AR	SP107	Ventana	RTU
CK7	OV-TL 12/30	Dako	1:800
HMGA2	D1A7	Cell signaling	1:100
MDM2	IF2	Thermo Scientific	1:100
PLAG1	3B7	Sigma	1:100
p40	DAK-p40	Dako	RTU
p63	DAKp63	Dako	RTU
S100	polyclonal	Dako	RTU
SOX10	SP267	Ventana	RTU

RTU denotes ready-to-use.

PLAG1 immunohistochemical examination was recognized as positive if moderate to strong nuclear staining was present at least focally (≥5% tumor cells). Cytoplasmic, membranous or non-specific diffuse granular staining was not considered positive. HMGA2 immunohistochemical stain was counted as positive only if strong diffuse nuclear staining was present.

### FISH

For *HMGA2* rearrangement detection performed in 24/40 (60%) cases, custom-designed probes with chromosomal locations: chr12:65821445-66218059 and chr12:66359930-66760219 were used (SureFish/Agilent Technologies). For the detection of *PLAG1* rearrangements performed in 18/40 (45%) cases, the probes SureFISH PLAG1 5’ BA 625 kb and SureFISH PLAG1 3’ BA 295 kb were used (SureFish/Agilent Technologies). FISH analysis was performed and interpreted as described elsewhere.^5^ In addition, *MDM2* enumeration FISH analysis was done in 5/40 (12.5%) cases as previously described.^6^

### Targeted Sequencing

For targeted RNA-sequencing, which was used in 39/40 (97.5%) cases, the customized Sarcoma or Solid Tumor FusionPlex kits (ArcherDX Inc.) were used. Final libraries were sequenced on NovaSeq. 6000 sequencer (Illumina), and Archer Analysis software were recruited to analyze the sequencing results (v6-8; ArcherDX Inc.). Fusion detection settings were adjusted to require at least 5 valid fusion reads with at least 3 distinct start sites among the valid fusion reads.

In addition, the mutation status of 2 cases was assessed using the commercially available TruSight Oncology 500 panel (Illumina). The OmnomicsNGS analysis program was used for data analysis (DNA variant filtering and annotation) (Euformatics, Finland). Reported variants were filtered retaining variations with coding effects, read depths >50, and variants with allelic frequency >10%, with the removal of benign variants according to the ClinVar database.^7^ The remaining variations were visually verified in raw data, and probable artifactual variants were eliminated.^8^

## RESULTS

### Demographic and Clinical Characteristics

The clinicopathologic data for all cases are summarized in Table 2, whereas further data for the genetic alterations detected are available in Supplementary File 1, Supplemental Digital Content 1, http://links.lww.com/PAS/C43. All patients were adults, their ages ranging from 22 to 95 years (mean=63, median=66.5). There was a slight female predilection. All cases represented primary tumors, most commonly occurring in the parotid gland (n=34), whereas 2 cases arose from small salivary glands of the lip and 1 case each originated from the submandibular gland, a small salivary gland in the parapharyngeal space, buccal mucosa and a peribronchial minor salivary gland-type tissue. Follow-up information was obtained in 7 cases with an average length of 34 months (ranging from 1 to 55 mo). All 7 patients were treated with surgical removal of the tumor only and had no evidence of disease at the end of the observation period.

**TABLE 2:** Main Clinicopathologic and Molecular-genetic Data.

No.	Sex	Age	Location	IHC								Fusion	FISH		Phasicity	Morphology notes	Outcome (mo)
				HMGA2	PLAG1	S100	SOX10	CK7	p63	p40	AR		HMGA2	PLAG1			
1	F	72	Parotid	+	−	+	+	+	+	−	−	*HMGA2::MSRB3*	ND	ND	Monophasic	Component of classic PA	
2	F	57	Parotid	+	−	+	+	−	Fo+	−	ND	*HMGA2::WIF1*	-	ND	Monophasic	Component of classic PA	
3	M	55	Parotid	+	−	+	+	+	−	−	ND	*HMGA2::LINCO2389*	+	ND	Monophasic		
4	F	77	Parotid	+	−	ND	+	+	+ (AB)	+ (AB)	+	ND	+	-	Biphasic		
5	M	63	Parotid	+	−	+	+	+	−	−	Fo+	*HMGA2::WIF1*	ND	ND	Monophasic		
6	M	66	Parotid	+	ND	+	+	+	−	−	−	*HMGA2::WIF1*	ND	ND	Monophasic		NED (41)
7	F	51	Parotid	+	−	+	+	+	Fo+	−	−	*HMGA2::WIF1*	ND	-	Monophasic	Component of classic PA	
8	M	46	Parotid	+	ND	+	+	+	−	−	−	*HMGA2::WIF1*	ND	ND	Monophasic		NED (49)
9	M	88	Parotid	+	−	+	+	+	−	−	Fo+	*HMGA2::WIF1*	ND	ND	Monophasic	Component of classic PA	
10	F	65	Parotid	+	−	+	+	+	−	−	−	−	+	-	Monophasic		
11	F	43	Parotid	+	−	+	+	+	−	−	ND	*HMGA2::WIF1*	ND	ND	Monophasic		
12	M	71	Parotid	+	−	+	+	+	−	−	ND	*HMGA2::WIF1*	ND	ND	Monophasic	Component of classic PA	NED (55)
13	M	75	Parotid	ND	−	+	+	+	−	−	ND	*HMGA2::ARID2*	+	ND	Monophasic		
14	F	57	Parotid	+	−	+ (AB)	+ (AB)	+ (L)	+ (AB)	+ (AB)	+	*HMGA2::LINC02389*	+	ND	Biphasic	Transformation into E-MCa	
15	F	76	Parotid	+	−	+	+	+	Fo+	−	ND	*HMGA2::WIF1*	+	-	Monophasic		NED (37)
16	M	59	Pharyngeal MSG	+ (AB)	−	+ (AB)	+	+ (L)	+ (AB)	ND	+	*HMGA2::FHIT*	-	-	Biphasic	Component of classic PA	NED (1)
17	M	71	Parotid	+	−	+	+	+	−	ND	ND	*HMGA2::WIF1*	-	NA	Monophasic		
18	F	64	Parotid	+	ND	+	+	+	−	ND	ND	*HMGA2::WIF1*	+	-	Monophasic	Component of classic PA	NED (24)
19	F	77	Parotid	+	−	ND	+	+	Fo+	ND	ND	*HMGA2::WIF1*	-	NA	Monophasic	Component of classic PA	
20	F	79	Parotid	+	−	+	+	+	Fo+	−	ND	*HMGA2::LINC02389*	ND	ND	Monophasic		
21	F	69	Parotid	+ (AB)	+ (AB)	+ (AB)	+ (AB)	+ (L)	+ (AB)	+ (AB)	+	*HMGA2::LINC02389*	+	-	Biphasic		
22	F	22	Buccal MSG	+	−	+ (AB)	+ (AB)	+ (L)	+ (AB)	+ (AB)	Fo+	−	+	NA	Biphasic	Component of classic PA	NED (31)
23	F	72	Parotid	+	−	+	+	+ (L)	+ (AB)	+ (AB)	+	−	+	NA	Biphasic	Component of classic PA	
24	F	75	Parotid	+	−	+ (AB)	+ (AB)	+ (L)	+ (AB)	+ (AB)	Fo+	*HMGA2::LINC02389*	ND	ND	Biphasic		
25	F	69	Peribronchal MSG	+	−	ND	+ (AB)	+ (L)	+ (AB)	+ (AB)	−	−	+	-	Biphasic		
26	F	34	Parotid	+	+ (AB)	ND	+ (AB)	+ (L)	+ (AB)	+ (AB)	+	*HMGA2::WIF1*	ND	ND	Biphasic	Component of classic PA	
27	M	46	Parotid	+	−	+	+	+	−	−	ND	*HMGA2::WIF1*	-	ND	Monophasic		
28	M	68	SG	+	−	+ (AB)	+ (AB)	+ (L)	+ (AB)	+ (AB)	ND	*HMGA2::IFNG-AS1*	ND	ND	Biphasic		
29	M	71	Parotid	+	−	+	+	+	+	−	ND	*HMGA2::WIF1*	-	ND	Monophasic		
30	F	95	Parotid	+	−	+	+	+	−	−	ND	*HMGA2::MSRB3*	+	ND	Monophasic		
31	M	65	Lip MSG	+	−	+ (AB)	+ (AB)	+ (L)	+ (AB)	+ (AB)	+	−	+	ND	Biphasic		
32	F	60	Lip MSG	+	−	+ (AB)	+ (AB)	+ (L)	+ (AB)	+ (AB)	+	*HMGA2::LINC02231*	ND	ND	Biphasic		
33	F	23	Parotid	+	−	+ (AB)	+ (AB)	+ (L)	+ (AB)	+ (AB)	+	−	+	-	Biphasic	Component of classic PA	
34	M	22	Parotid	+	−	ND	+ (AB)	+ (L)	+ (AB)	+ (AB)	+	−	+	-	Biphasic		
35	F	66	Parotid	+	−	+	+	+	Fo+	ND	ND	*HMGA2::WIF1*	ND	ND	Monophasic		
36	M	73	Parotid	+ (AB)	−	+ (AB)	+ (AB)	+ (L)	+ (AB)	+ (AB)	+	−	+	-	Biphasic	Component of classic PA	
37	F	81	Parotid	+	−	+	+	+	−	−	ND	*HMGA2::WIF1*	-	-	Monophasic		
38	F	56	Parotid	+	−	+	+	+	−	−	ND	−	+	NA	Monophasic	Component of classic PA	
39	F	67	Parotid	+	−	+	+	+	Fo+	−	ND	*HMGA2::WIF1*	ND	ND	Monophasic		
40	F	69	Parotid	ND	−	+	+	+	−	ND	ND	*HMGA2::MSRB3-AS1*	ND	NA	Monophasic		

− indicates negative; +, positive; AB, abluminal tumor cells; E-MCa, epithelial-myoepithelial carcinoma; F, female; Fo, focally; L, luminal tumor cells; M, male; MG, mucous gland; MSG, minor salivary gland; NA, not analyzable; ND, not done; NED, no evidence of disease; PA, pleomorphic adenoma; SG, submandibular gland.

### Histopathologic and Molecular-genetic Features

**FIGURE 1:**
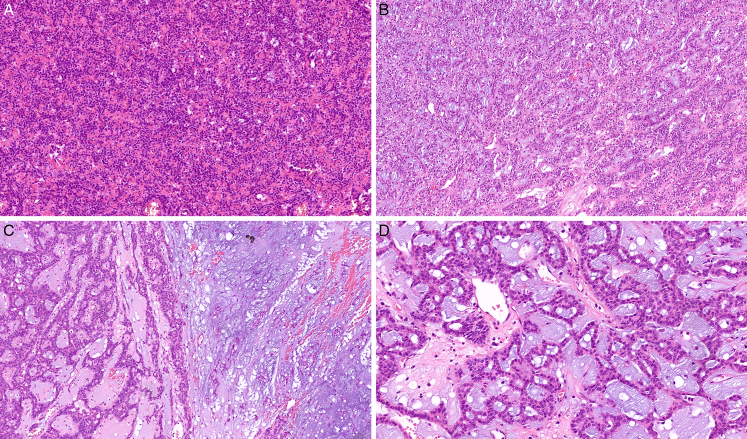
Histomorphologic features of monophasic canalicular adenoma-like tumors. A, Hypercellular proliferation of uniform epithelial cells growing in interconnected canaliculi and trabeculae, within a sclerotic stroma (case 5). B, Less dense, fibromyxoid stroma was present in some cases (case 3). C, A component of conventional pleomorphic adenoma was present in 8 monophasic cases (case 2). D, Abundant loose myxoid stroma was present in case 2, creating a pseudomicrocystic appearance in some areas.

**FIGURE 2:**
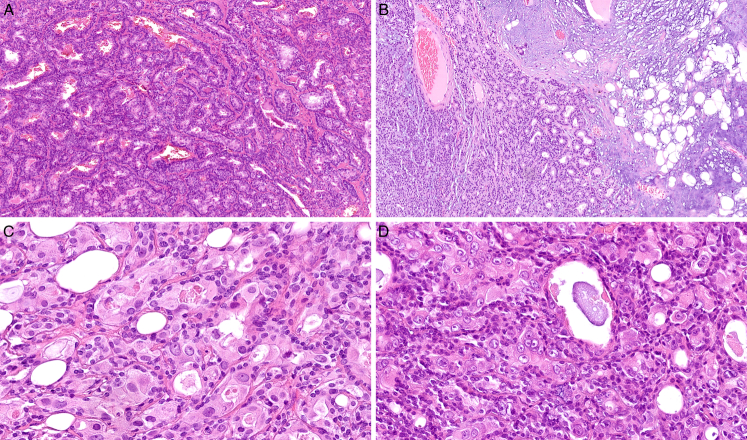
Histomorphologic features of biphasic canalicular adenoma-like tumors. A, A highly cellular biphasic proliferation of intertwined canaliculi and tubules (case 30). B, A component of conventional pleomorphic adenoma was present in 6 biphasic cases (case 26). C, Apocrine change of the luminal cells with nuclear enlargement, conspicuous nucleoli and abundant pale eosinophilic cytoplasm was present at least focally in all biphasic cases (case 4). D, Detail of the nuclear atypia in the luminal cells with apocrine change (case 36).

All cases exhibited a distinctive growth pattern consisting of interconnected and confluent trabeculae and canaliculi lined with monolayered to multilayered epithelium (Figs. 1–2). The hypocellular stroma varied in appearance from dense hyalinized collagenous (Figs. 1A, 2A) to completely myxoid areas (Figs. 1B, D) and contained a network of thin-walled capillaries. Out of 37 assessable cases, most tumors (n=35) were encased by at least partial fibrous pseudocapsule, being strikingly thick in 3 cases. Two cases grew as a cohesive circumscribed lesion but devoid of any pseudocapsule. In 7 cases, satellite nodules were observed. In addition, 10 cases exhibited denser cellular foci with solid nests (Figs. 1A, 2A) and 8 cases contained cystic or microcystic areas (Fig. 1D). A component of conventional PA was revealed in 14/40 (35%) of cases (Figs. 1C, 2B) either as separate PA nodules (n=4) or admixed with the canalicular tumor (n=10). Eight of these PA-associated cases had monophasic morphology (8/25; 32% of the monophasic tumors) (Fig. 1C) whereas the remaining 6 cases were biphasic (6/15; 40% of the biphasic tumors) (Fig. 2B).

**FIGURE 3:**
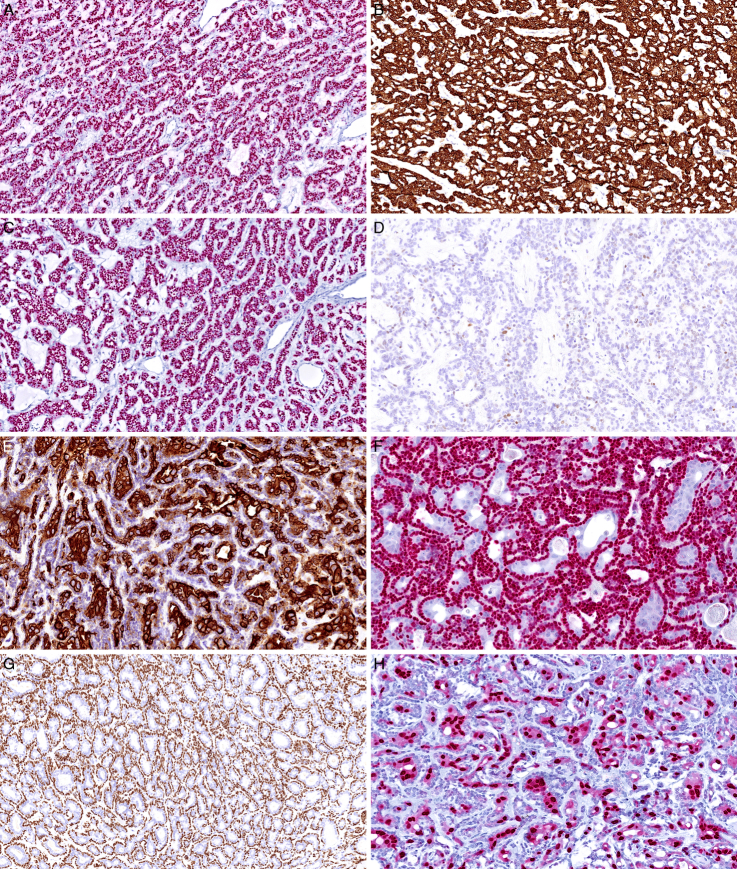
Immunohistochemical features of the tumors. A, HMGA2 immunostaining was positive in all cases (case 3). B, The monophasic cases stained diffusely with CK7 (case 3). C, All cells of the monophasic tumors were positive for SOX10 (case 3). D, Scattered and focal positivity for p63 was detected in one-third of the monophasic cases. E, In biphasic cases, CK7 was usually positive in the luminal cells only (case 36). F, SOX10 highlighted the abluminal cells in the biphasic cases (case 36). G, p63 stained the nuclei of the abluminal cells in the biphasic case (case 26). H, Luminal cells in the biphasic cases displayed apocrine change with strong nuclear immunopositivity for AR (case 36).

Overall, the immunohistochemical surrogate marker HMGA2 was positive in 100% of the analyzed cases (Fig. 3A). In 3 cases with biphasic morphology, HMGA2 was only detected in the abluminal layer of tumor cells. PLAG1 was immunohistochemically negative in all analyzed cases. S100 and SOX10 immunoexpression was detected in all cases, whereas CK7 was positive in 39/40 (97.5%) cases.

Twenty-five tumors (62.5%) exhibited a monophasic pattern (Fig. 1), being composed mostly of small epithelial cells with a moderate amount of eosinophilic cytoplasm and round to oval nuclei without striking atypia or conspicuous nucleoli (Fig. 1A-B). One case was composed of basaloid-appearing cells with focal nuclear palisading. The epithelium appeared monolayered to multilayered in the areas where the trabeculae fused or divided. Tumors with more abundant myxoid stroma focally exhibited pseudomicrocystic pattern (Fig. 1C-D). All tumor cells were immunopositive for S100 and SOX10 (Fig. 3C), and all but one cases stained with the CK7 antibody (Fig. 3B). Although p40 was completely negative in all cases tested, p63 exhibited scattered limited immunopositivity in 8/25 (32%) monophasic cases (Fig. 3D) and was diffusely positive in 1 case (4%). Focal AR-immunopositivity was detected in 2/25 (8%) monophasic cases. All 3 monophasic cases that were tested for MDM2 protein expression by immunohistochemistry were completely negative. In addition, FISH did not reveal any quantitative changes in the *MDM2* gene in these 3 cases.

A biphasic pattern was noted in 15 cases (37.5%), comprising mostly bilayered to multilayered trabeculae of flattened abluminal and cuboidal to columnar luminal cells (Fig. 2). Although the abluminal cells stained positively with p40 and p63 antibodies (Fig. 3G), the luminal cells were negative. CK7 was positive in the luminal cells in 14/15 (93%) biphasic cases (Fig. 3E), and one biphasic tumor displayed CK7 immunopositivity in both luminal and abluminal cells. S100 and SOX10 (Fig. 3F) were usually positive in the abluminal cells; however, in 1 and 3 cases, respectively, both cell populations displayed immunopositivity with these antibodies. All biphasic tumors showed focal to diffuse apocrine transformation of the luminal cells. These cells had pale eosinophilic, focally finely granular cytoplasm and variably sized nuclei with occasional conspicuous deeply eosinophilic nucleoli (Fig. 2C-D). Multifocal to diffuse nuclear AR-immunopositivity was revealed in the apocrine cell population in 13/14 (93%) of tested biphasic cases (Fig. 3H). Both biphasic cases that were tested were immunonegative for MDM2 protein expression and no quantitative changes of the *MDM2* gene were detected by FISH.

**FIGURE 4:**
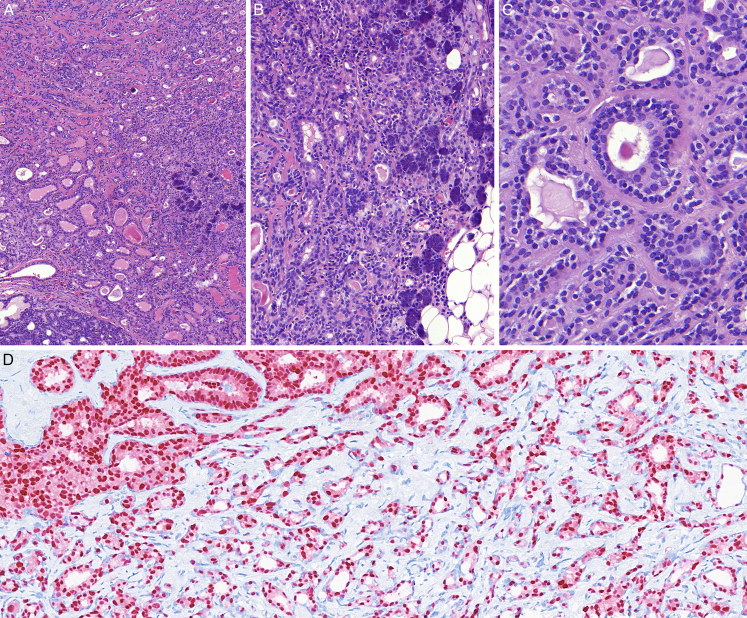
An invasive component in case 14, seemingly arising from the biphasic canalicular adenoma-like subtype of pleomorphic adenoma, was diagnosed as epithelial-myoepithelial carcinoma. A, Residual canalicular adenoma-like pleomorphic adenoma (right side) and the invasive tumor retaining the biphasic canalicular and tubular growth patterns but growing invasively into the surrounding parotideal and adipose tissue. B, The invasive tumor infiltrating the parotideal acini and adjacent adipose tissue. C, Detail of the 2-layered microcystic/tubular pattern of growth. D, Nuclear immunopositivity for AR in the luminal cells of the invasive tumor.

The majority of the tumors grew as circumscribed lesions and did not show alarming atypia, allowing for a diagnosis of a benign salivary gland tumor. However, in case 14, a biphasic canalicular tumor transformed into a carcinoma infiltrating the surrounding adipose and parotideal tissue (Fig. 4A-C), composed of a less regular canalicular, microcystic and tubular proliferation of moderately atypical neoplastic cells with enlarged nuclei and pale eosinophilic cytoplasm. The epithelium still retained the biphasic morphology of the adjacent canalicular tumor with CK7- and AR-immunopositivity of the luminal cells (Fig. 4D). The abluminal cells displayed myoepithelial morphology with occasional cytoplasmic clearing and immunopositivity of myoepithelial markers (p40, p63, smooth muscle actin, calponin). In some areas, the myoepithelial layer became extremely flattened, inconspicuous and almost indiscernible on hematoxylin-and-eosin stains. Detection of *HRAS* mutation by NGS was attempted but the material was of insufficient quality for the analysis. Nevertheless, the malignant component was diagnosed as an epithelial-myoepithelial carcinoma based in morphology and immunoprofile.

All cases harbored an *HMGA2* rearrangement. As analyzed by FISH, the *HMGA2* break-apart was present in 17/24 (71%) cases. Overall, an *HMGA2* rearrangement without a fusion partner recognized by RNA-sequencing was present in 9 cases, and in 1 case where RNA-sequencing was not performed. The FISH-negative cases were included in the study because further analysis with targeted sequencing revealed *HMGA2::WIF1*, *HMGA2::MSRB3*, and *HMGA2::FHIT* fusions in 6 and 1 case each, respectively. A fusion involving *HMGA2* was detected in 30/39 (77%) cases tested by targeted sequencing, with the most common *HMGA2::WIF1* fusion being detected in 18 tumors. Other fusion partners to *HMGA2* included *ARID2*, *FHIT*, *MSRB3* and its antisense variant *MSRB3-AS1*, *IFNG-AS1*, and the long intergenic region *LINC02389*. *PLAG1* rearrangement was absent in all 12 cases analyzable by FISH, whereas the remaining 6 cases tested for this rearrangement were not analyzable. No correlation between the fusion partner and morphologic features was noted. The molecular-genetic findings of all 40 cases are summarized in Table 2.

## DISCUSSION

In this study, we present an extensive analysis of the immunohistochemical and molecular-genetic features of the emerging canalicular tumor of the salivary glands, defined by *HMGA2* rearrangement and a unique anastomosing canalicular and trabecular arrangement of monophasic or biphasic epithelium. The biphasic tumors displayed diffuse p40 and p63 immunopositivity in the abluminal cells, whereas the luminal cells were negative for these markers. In the monophasic tumors, p40 was completely negative but p63 stained at least scattered tumor cells in one-third of cases.

The biphasic tumors, which represented 37.5% of cases in this study, displayed at least focal apocrine transformation of the luminal cells, including nuclear enlargement and size variation with occasional conspicuous nucleoli, nuclear immunopositivity for AR, and increased amounts of finely granular pale eosinophilic cytoplasm. Although the luminal cells were completely negative for p40 and p63 and stained strongly with CK7, the abluminal cells displayed an opposite myoepithelial-like immunoprofile, with CK7 negativity and p40, p63, S100, and SOX10 positivity in all cases tested. A component of conventional PA was present in 6 biphasic cases.

Although all of the biphasic cases displayed at least a moderate degree of nuclear atypia, the MDM2 immunohistochemical staining performed in 2 of these cases was negative. In agreement with this finding, no *MDM2* amplification was revealed by FISH. Furthermore, 3 monophasic cases were tested by immunohistochemistry and FISH to detect amplification or overexpression of *MDM2* with negative results. The pronounced gear-like nuclear atypia reported to be associated with *MDM2* amplification in carcinoma ex PA^4^ might be a rare phenomenon and was not particularly recognizable in our cases.

Almost two-thirds of cases included in this study (62.5%) had a monophasic growth pattern, of cords and trabeculae of bland-looking epithelial cells. Notably, the tumor cells were consistently immunopositive for S100 and SOX10 as well as CK7, whereas p40 was completely negative in all cases tested and p63 exhibited only scattered positivity in 8/25 cases (32%) and diffuse positivity in 1/25 cases (4%). Similar to the biphasic tumors, the monophasic tumors contained a conventional PA component in 8 cases.

The canalicular tumors described herein likely represent a variant of PA. Not only do they share *HMGA2* rearrangements but in more than one-third of the cases, areas with the typical PA morphology were part of both the monophasic and biphasic neoplasms described in this study (in 32% and 40% of the monophasic and biphasic tumors, respectively). However, the canalicular adenoma-like subtype of PA differs from the conventional PA by both immunoprofile (with limited p63 immunopositivity and complete lack of p40 staining in the canalicular adenoma-like subtype) and the distinctive canalicular and trabecular growth pattern with strikingly higher cellularity than is seen in conventional PA.

In addition, one of our cases had transformed into an invasive epithelial-myoepithelial carcinoma with AR-immunopositive luminal tumor cells. This represents a 2.5% rate of malignancy arising in this variant of PA, which is considerably lower than the 21% rate reported by Katabi et al.^2^ The real incidence of malignant transformation is probably even lower, as most of the cases reported in the literature were selected to be reported based upon their known genetic characteristics. Unequivocally benign cases of canalicular adenoma-like PA are less likely to be tested with molecular-genetic methods and therefore omitted, introducing bias into the whole cohort.

All cases included in this study harbored an *HMGA2* gene rearrangement. Although an *HMGA2* break-apart without a gene fusion partner was detected by FISH in about one-quarter of the cases described herein, a fusion involving *HMGA2* was detected in 30/39 (77%) cases tested by targeted RNA-sequencing. The *HMGA2::WIF1* was the most common fusion, whereas the other fusion partners to *HMGA2* included *ARID2*, *FHIT*, *MSRB3* and its antisense variant *MSRB3-AS1*, *IFNG-AS1*, and the long intergenic region *LINC02389*. Whereas the *HMGA2::MSRB3* and *HMGA2::FHIT* fusions were described in previous studies,^3,9^ the remaining detected fusion partners such as *ARID2* or *IFNG-AS1* are novel in this tumor type. All of the fusion partners were located in the vicinity of the *HMGA2* gene on the long arm of chromosome 12, some of them representing non-classical partner genes coding for regulatory RNAs. Altogether, the specific partner is likely not determinative of the action of the fusion product, especially as we also detected some cases without an apparent fusion partner to *HMGA2*. The common denominator for the aforementioned rearrangements is the loss of the 3’-untranslated region of the *HMGA2* gene, which plays an important role in the negative regulation of HMGA2 activity. In an experimental in vitro setting, binding of microRNA *let-7* to the 3’-untranslated region promoted degradation of *HMGA2* RNA, and the growth of lung cancer cells was inhibited by transfection of *let-7*.^10,11^ Notably, in 12 canalicular adenomas tested by Agaimy et al,^1^ neither *HMGA2* fusions (0/4) nor HMGA2 immunoreactivity (0/12) was detected.

In summary, we present further insight into the genetic background of an emerging canalicular adenoma-like subtype of PA. All cases in this study harbored an *HMGA2* rearrangement, detected by FISH or targeted RNA-sequencing. A fusion involving *HMGA2* was detected in 30/39 (77%) cases tested by targeted sequencing, most commonly being represented by the *HMGA2::WIF1*, whereas the other novel or rare fusion partners to *HMGA2* included *ARID2*, *FHIT*, *MSRB3* and its antisense variant *MSRB3-AS1*, *IFNG-AS1*, and the long intergenic region *LINC02389*. A common hallmark of the genetic alterations detected in the cases described herein was the loss of the 3’-untranslated region of *HMGA2*, which, when retained, enables negative regulation of HMGA2 signaling. Furthermore, we provide additional information about the lesion’s morphological and immunohistochemical features, describing its monophasic or biphasic growth patterns with limited p63 immunopositivity and complete lack of p40 immunostaining, as well as the AR-immunopositive apocrine luminal cells in the biphasic tumors.

## Supplementary Material

## References

[1] Agaimy A Ihrler S Baneckova M. HMGA2-WIF1 rearrangements characterize a distinctive subset of salivary pleomorphic adenomas with prominent trabecular (canalicular adenoma-like) morphology. Am J Surg Pathol. 2022;46:190–199. 34324456 10.1097/PAS.0000000000001783

[2] Katabi N Sukhadia P DiNapoli SE. Expanding the histological spectrum of salivary gland neoplasms with HMGA2::WIF1 fusion emphasising their malignant potential: a report of eight cases. Histopathology. 2024;84:387–398. 37849332 10.1111/his.15074 PMC10841865

[3] Alsugair Z Lepine C Descotes F. Beneath HMGA2 alterations in pleomorphic adenomas: Pathological, immunohistochemical, and molecular insights. Hum Pathol. 2024;152:105633. 39089476 10.1016/j.humpath.2024.105633

[4] Alsugair Z Fieux M Descotes F. Peculiar nuclear atypia associated with MDM2 gene amplification in carcinoma ex-pleomorphic adenoma harbouring an alteration of HMGA2. Histopathology. 2024;85:338–346. 38708906 10.1111/his.15209

[5] Steiner P Andreasen S Grossmann P. Prognostic significance of 1p36 locus deletion in adenoid cystic carcinoma of the salivary glands. Virchows Arch. 2018;473:471–480. 29619555 10.1007/s00428-018-2349-6

[6] Santana T Pavel A Martinek P. Biomarker immunoprofile and molecular characteristics in salivary duct carcinoma: clinicopathological and prognostic implications. Hum Pathol. 2019;93:37–47. 31437521 10.1016/j.humpath.2019.08.009

[7] Landrum MJ Lee JM Benson M. ClinVar: improving access to variant interpretations and supporting evidence. Nucleic Acids Res. 2018;46(D1):D1062–D1067. 29165669 10.1093/nar/gkx1153 PMC5753237

[8] Koboldt DC. Best practices for variant calling in clinical sequencing. Genome Med. 2020;12:91. 33106175 10.1186/s13073-020-00791-w PMC7586657

[9] Geurts JM Schoenmakers EF Röijer E. Expression of reciprocal hybrid transcripts of HMGIC and FHIT in a pleomorphic adenoma of the parotid gland. Cancer Res. 1997;57:13–17. 8988031

[10] Lee YS Dutta A. The tumor suppressor microRNA let-7 represses the HMGA2 oncogene. Genes Dev. 2007;21:1025–1030. 17437991 10.1101/gad.1540407 PMC1855228

[11] Borrmann L Wilkening S Bullerdiek J. The expression of HMGA genes is regulated by their 3’UTR. Oncogene. 2001;20:4537–4541. 11494149 10.1038/sj.onc.1204577

