# Supplementary material for: Expanding the Molecular-genetic Spectrum of Canalicular Adenoma-like Subtype of Pleomorphic Adenoma of Salivary Glands
Source: Am J Surg Pathol. 2025 Mar 4;49(6):554–63. doi: 10.1097/PAS.0000000000002377 (PMC12068546; doi:10.1097/PAS.0000000000002377)
Supplement: Supplementary file 1 [file pas-49-554-s001.docx]

**Supplementary File 1** Details of the molecular-genetic alterations

| No. | Fusion | Chromosome breakpoints (reference genome GRCh37) | FISH | |
| --- | --- | --- | --- | --- |
|  |  |  | HMGA2 | PLAG1 |
| 1 | *HMGA2::MSRB3* | chr12:66232349,chr12:65856935 | ND | ND |
| 2 | *HMGA2::WIF1* | chr12:66345195, chr12:65449906 | - | ND |
| 3 | *HMGA2::LINCO2389* | chr12:66357152,chr12:65368351 | + | ND |
| 4 | ND |  | + | - |
| 5 | *HMGA2::WIF1* | chr12:66232349,chr12:65445250 | ND | ND |
| 6 | *HMGA2::WIF1* | chr12:66232349,chr12:65449906 | ND | ND |
| 7 | *HMGA2::WIF1* | chr12:66357152,chr12:65471634 | ND | - |
| 8 | *HMGA2::WIF1* | chr12:66232349,chr12:65445250 | ND | ND |
| 9 | *HMGA2::WIF1* | NA | ND | ND |
| 10 | - |  | + | - |
| 11 | *HMGA2::WIF1* | chr12:66232349,chr12:65445250 | ND | ND |
| 12 | *HMGA2::WIF1* | chr12:66357152,chr12:65471634 | ND | ND |
| 13 | *HMGA2::ARID2* | chr12:66232349,chr12:46298717 | + | ND |
| 14 | *HMGA2::LINC02389* | chr12:66232349,chr12:65368351 | + | ND |
| 15 | *HMGA2::WIF1* | chr12:66232349,chr12:65445250 | + | - |
| 16 | *HMGA2::FHIT* | chr12:66232349,chr3:59738047 | - | - |
| 17 | *HMGA2::WIF1* | chr12:66232349,chr12:65448993 | - | NA |
| 18 | *HMGA2::WIF1* | chr12:66232349,chr12:65456356 | + | - |
| 19 | *HMGA2::WIF1* | chr12:66232349,chr12:65448993 | - | NA |
| 20 | *HMGA2::LINC02389* | chr12:66345195,chr12:65368351 | ND | ND |
| 21 | *HMGA2::LINC02389* | chr12:66345195,chr12:65368351 | + | - |
| 22 | - |  | + | NA |
| 23 | - |  | + | NA |
| 24 | *HMGA2::LINC02389* | chr12:66232349,chr12:65368351 | ND | ND |
| 25 | - |  | + | - |
| 26 | *HMGA2::WIF1* | chr12:66232349,chr12:65445250 | ND | ND |
| 27 | *HMGA2::WIF1* | chr12:66232349,chr12:65448993 | - | ND |
| 28 | *HMGA2::IFNG-AS1* | chr12:66232349,chr12:68413642 | ND | ND |
| 29 | *HMGA2::WIF1* | chr12:66232349,chr12:65445250 | - | ND |
| 30 | *HMGA2::MSRB3* | chr12:66345195, chr12:65702309 | + | ND |
| 31 | - |  | + | ND |
| 32 | *HMGA2::LINC02231* | chr12:66345195,chr12:65351637 | ND | ND |
| 33 | - |  | + | - |
| 34 | - |  | + | - |
| 35 | *HMGA2::WIF1* | chr12:66232349,chr12:65448993 | ND | ND |
| 36 | - |  | + | - |
| 37 | *HMGA2::WIF1* | chr12:66232349,chr12:65449891/ chr12:65449958/chr12:65445250 | - | - |
| 38 | - |  | + | NA |
| 39 | *HMGA2::WIF1* | chr12:66232349,chr12:65445250 | ND | ND |
| 40 | *HMGA2::MSRB3-AS1* | chr12:66232349,chr12:65963828 | ND | NA |

Abbreviations: NA denotes not-analyzable/not-available; ND, not done; +, positive; -, negative.
